# Effect of implementing a birth plan on maternal and neonatal outcomes: a randomized controlled trial

**DOI:** 10.1186/s12884-022-05199-5

**Published:** 2022-11-22

**Authors:** Parivash Ahmadpour, Sanaz Moosavi, Sakineh Mohammad-Alizadeh-Charandabi, Shayesteh Jahanfar, Mojgan Mirghafourvand

**Affiliations:** 1 Students’ Research Committee, Midwifery Department, Faculty of Nursing and Midwifery, Tabriz University of Medical Sciences, Tabriz, Iran; 2 Women Reproductive Health Research Center, Tabriz University of Medical Sciences, Tabriz , Iran; 3 Midwifery Department, Faculty of Nursing and Midwifery, Tabriz University of Medical Sciences, Tabriz, Iran; 4 Public Health Department, Central Michigan University, Michigan, USA; 5 Social Determinants of Health Research Center, Tabriz University of Medical Sciences, Tabriz , Iran

**Keywords:** Birth Plan, Childbirth Experiences, Randomized Controlled Trial, Labor

## Abstract

**Background:**

The birth plan is an approach for pregnant women to offering their expectations of labor and birth. The purpose of this study was to investigate the effect of birth plan on maternal and neonatal outcomes.

**Methods:**

This study was a randomized controlled clinical trial performed on 106 pregnant women, 32–36 weeks of pregnancy, referring to Taleghani educational hospital in Tabriz city-Iran. Participants were randomly assigned to the two groups of birth plan and control using a randomized block method. Participants in the birth plan group received the interventions based on the mother's requested birth plan. The birth plan included items of the mother's preferences in labor, mobility, eating and drinking, monitoring, pain relief, drug options, labor augmentation, pushing, amniotomy, episiotomy, infant care, and caesarean section. The control group received routine hospital care. The primary outcomes were childbirth experience and duration of the active phase of labor and the secondary outcomes were support and control in labor, fear of labor, post-traumatic stress disorder (PTSD), postpartum depression, duration of the second and third phases of labor, frequency of vaginal delivery, frequency of admission of newborn in NICU (Neonatal Intensive Care Unit), the mean first and fifth minute Apgar scores. The socio-demographic and obstetrics characteristics questionnaire, Wijma Delivery Expectancy/Experience Questionnaire (W-DEQ-versions A), and Edinburgh Postnatal Depression Scale (EPDS) were completed at the beginning of the study (at the gestational age of 32–36 weeks). The questionnaire of delivery information, neonatal information, and Delivery Fear Scale (DFS) was completed during and after the delivery. Also, a partogram was completed for all participants by the researcher. The participants in both groups followed up until 4–6 weeks post-delivery, whereby the instruments of Childbirth Experience Questionnaire 2.0 (CEQ2.0), Support and Control In Birth (SCIB) scale, EPDS, and PTSD Symptom Scale 1 (PSS-I) were completed by the researcher through an interview. The independent t-test, the chi-square test, and ANCOVA was used to analyze.

**Results:**

The mean (SD) of CEQ score was singificnalty higher in in the birth plan group (3.2 ± 0.2) compared to the control (2.1 ± 0.2) (MD = 1.0; 95% CI: 1.1 to 0.9; *P*˂0.001). Also, the mean (SD) SCIB score in the birth plan group was significantly higher than that of those in the control group (*P*˂0.001). The mean scores of DFS (*P* = 0.015), EPDS (*P*˂0.001), and PTSD (*P*˂0.001) as well as the frequency of emergency caesarean section (*P* = 0.007) in the birth plan group were significantly lower than those in the control group.

**Conclusion:**

This was the first study to assess the implementation of a birth plan in Iran. Based on the findings, a birth plan improves childbirth experiences; increases perceived support and control in labor; reduces fear of delivery; suppresses psychological symptoms of depression and PTSD, and increases the frequency of vaginal delivery.

Trial registration.

Iranian Registry of Clinical Trials (IRCT): IRCT20120718010324N58. Date of registration: 07/07/2020; URL: https://en.irct.ir/trial/47007; Date of first registration: 19/07/2020.

**Supplementary Information:**

The online version contains supplementary material available at 10.1186/s12884-022-05199-5.

## Background

The experiences that women gain during the delivery process are considered major childbirth outcomes. These outcomes affect mothers throughout their lives [1]. A positive childbirth experience implies that a woman is satisfied with the care and support she receives during pregnancy, childbirth, and postpartum and feels that she and her baby are the centers of attention and care [2]. Childbirth experiences lead to several short-term and long-term psychological outcomes. Despite the serious complications of childbirth, mothers who receive proper support and care during labor will enjoy positive childbirth experiences. These experiences will remain in the minds of mothers for years [3].

Negative childbirth experiences are associated with issues such as fear of childbirth, decreased desire for pregnancy, increased tendency for elective cesarean section (C-section) in future pregnancies, reduced quality of life, post-traumatic stress disorder (PTSD), and postpartum depression [4, 5]. These disorders adversely affect women and their interpersonal relationships. They are also associated with poor neonatal outcomes and cause cognitive and behavioral problems in children. In addition, these problems can negatively affect a ' 'woman's relationship with her partner. Mental health disorders of mothers substantially influence themselves as well as their families [6]. According to the World Health Organization (WHO), mental illnesses are one of the main indirect causes of all maternal mortality occurring within a year after birth; therefore, the issue of maternal mental health has been integrated into maternal and neonatal health care plans [7].

Several factors can help mothers undergo a positive childbirth experience. Participation of mothers in the decision-making process and their perceived control during childbirth are among these factors. In a systematic review, providing continuous support and paying attention to the needs of mothers during labor and delivery were identified as the main determinants of childbirth experience [4]. The positive relationship of the pregnant mother with the health care provider as well as her participation in the decision-making process can increase her satisfaction with the delivery process. Women who participate in the self-care process have a greater sense of control; thus, they often enjoy a positive childbirth experience [8]. Childbirth experience is also influenced by various social, environmental, organizational, and policy factors. Moreover, a good midwife-mother relationship can help mothers undergo positive childbirth experiences by reinforcing the perceived control of mothers over childbirth events and processes [9].

The idea of the birth plan was first introduced by Simkin and Reinke in 1980 as part of childbirth education class curriculum out of a generation where birth had become medicalized and choices for women had become very limited [10, 11]. It was a great movement aimed at reclaiming ' 'women's and 'patients' rights and allowing women to express their expectations and needs regarding the childbirth process [12]. The birth plan includes a ''woman's preferences for managing her labor. It helps them enjoy a better childbirth experience by gaining more control over their labor [13]. Most women who write their birth plans demand a drug-free delivery with few interventions [11]. This plan is primarily a tool for educating and empowering women, encouraging joint decision-making, facilitating communication, and building trust between mothers and their caregivers [12]. Despite the heterogeneity of birth plans, birth plans when developed in collaboration with care providers were associated with positive outcomes for childbearing women, and it may be used as an effective tool for increasing physiological birth rate, improving communication with health staff, controlling the labor and delivery processes, improving maternal and neonatal outcomes, and increasing delivery satisfaction [14].

Only a handful of clinical trials have been carried out to investigate the effects of the birth plan on maternal and neonatal outcomes and women's birth experiences [15, 16]. Furthermore, the results of a systematic review (2018) reveal the poor quality of the conducted studies as well as the inadequacy of existing evidence on the impact of the birth plan on ' 'women's childbirth experiences [17]. Unlike developed countries, a birth plan is a new issue in developing countries [15]. As far as the auhtors are aware, no birth plan has so far been implemented in Iran.

Given the high prevalence of C-section in Iran, Iranian ' 'women's fear of vaginal delivery, their poor participation in labor and delivery, and their negative experiences of vaginal delivery, studies must be carried out to improve women's childbirth experiences, enhance maternal and neonatal outcomes, and increase women's active involvement in labor and delivery [18]. Therefore, this study aimed to investigate the effects of the birth plan on maternal and neonatal outcomes.

## Assessed outcomes

The primary outcomes were childbirth experience and duration of the active phase of labor and the secondary outcomes were support and control in labor, fear of labor, post-traumatic stress disorder (PTSD), postpartum depression, duration of the second and third phases of labor, frequency of vaginal delivery, frequency of admission of newborn in NICU, the mean first and fifth minute Apgar scores.

## Study hypotheses

### Hypotheses for primary outcomes

1. The average score of childbirth experiences of women in the birth plan group is higher than the control group.
2. The average score of childbirth experiences of women in the birth plan group is higher than the control group.

### Hypotheses for secondary outcomes

1. The average duration of the second stage of labor in the birth plan group is less than the control group.
2. The average duration of the third stage of labor in the birth plan group is less than the control group.
3. The average score of control and support during childbirth in the birth plan group is higher than the control group.
4. The average score of fear of childbirth in the birth plan group is lower than the control group.
5. The frequency of spontaneous vaginal delivery is higher in the birth plan group than in the control group.
6. The average post-traumatic stress score in the birth plan group is lower than the control group.
7. The frequency of hospitalization of newborn in the NICU in the birth plan group is lower than in the control group.
8. The average postpartum depression score in the birth plan group is lower than the control group.
9. The average newborn Apgar score in the first and fifth minutes of birth in the birth plan group is higher than the control group.

 

## Methods

### Study design

In this randomized, parallel group, two arm, superiority trial with two parallel group, the researcher enrolled 102 pregnant women at the gestational age of 32–36 weeks visiting the Midwifery Clinic of Taleghani Medical Research and Training Hospital from late November 2020 to late June 2021. The protocol of the study has already been published [19].

### Setting

The research environment in this study was Taleghani Educational-Therapeutic Center in Tabriz-Iran. This hospital has a specialized gynecology and obstetrics clinic, general surgery, neonatal, NICU, labor and delivery, and high-risk pregnancy departments. On average, 200 births are performed in this hospital every month. Births in this hospital are performed by midwifery students under the supervision of midwifery instructors and professors and obstetrician residents. *participants.*

All 18 years old or older literate women at the gestational age of 32–36 weeks with a single fetus and a depression score < 13, who were living in Tabriz and were planning to have their first or second vaginal delivery at the Taleghani Hospital were included. The exclusion criteria included non-cephalic presentations, indications for C-section (*e.g.,* abnormal presentation, placenta previa, etc*.*), obstetric problems (*e.g.,* placenta previa, post-C-section vaginal delivery, placental abruption, and preeclampsia), high-risk pregnancies (*e.g.,* diabetes, heart diseases, etc*.*), stillborn, and abnormal fetus.

### Sample size

The sample size in this study was calculated based on the two variables of "childbirth experience"" and "duration of the active phase of labor" using G-power software. Based on the results of the study by Ghanbari Homaei et al. [20] on the variable of childbirth experience and considering m_1_ = 2.71, m_2_ = 3.25 (assuming 20% increase in response to the intervention), sd_1_ = sd_2_ = 0.73, one-sided α = 0.05, and power = 90%, the sample size was calculated in 32 participants; considering 10% attrition, the sample size was considered 35 in each group. According to the variable of the duration of the active phase of labor and concerning m_1_ = 276.7, m_2_ = 221.4 (assuming 20% reduction in response to intervention), sd_1_ = sd_2_ = 91.3, α = 0.05, and power = 90%, it was calculated as 48 participants, and eventually, it will be 53 as the sample size in each group, given 10% possible attrition. Since the sample size calculated based on the variable of the duration of the active phase of labor was larger than that of childbirth experience, the final sample size was considered 53 in each group.

### Sampling

The ethics committee approved this studyof Tabriz University of Medical Sciences (Ethics code: IR.TBZMED.REC.1399.278). After registering the study at Iranian Registry of Clinical Trials (IRCT) (Registry code: IRCT20120718010324N58), the researcher used convenience sampling to select the sample among all eligible women visiting the Midwifery Clinic of Taleghani Hospital. The participants then completed the Edinburgh Postnatal Depression Scale (EPDS). It should be noted that pregnant women with EPDS scores ≥ of 13 were referred to a psychiatrist. Explanations were provided to the participants about the research method and objectives. Written informed consent was obtained from those who were willing to participate in the study. The participants then completed the socio-demographic and obstetrics characteristics questionnaire, the Wijma Delivery Expectancy/Experience Questionnaire-version A (W-DEQ-version A).

### Randomization and allocation concealment

A randomized block method with 4- and 6-blocks was used to allocate the participants to the studied groups with an allocation ratio of 1:1. For allocation concealment, the type of intervention is written on a piece of paper and was placed inside consecutively numbered opaque envelopes. After the study participants signed the consent form, the relevant envelope was opened, and the intervention was implemented. Random allocation was conducted by a person not involved in the sampling and data collection.

### Intervention and follow-up

After assigning the women to the intervention and control groups, those in the intervention group attended a training session thoroughly. An educational booklet explaining all items of the birth plan checklist was also provided to the participants. In addition, the ' 'researcher's phone number was given to the mothers to ask their relevant questions. Two weeks later, the mothers attended another session to develop the birth plan. Both sessions was conducted by the the principal investigator (first author; PhD student of Midwifery) under supervision of an obstetrician. All women attended at both sessions. The plan developed by each participant was revised and approved by an obstetrician.

The researcher contacted the mothers by phone or in-person (at the request of mothers) in the interval between the two face-to-face sessions. Moreover, the participants were also asked to contact the researcher whenever they arrived at the hospital for delivery. With the entrance of the mothers to the labor ward, the birth plan was implemented by the researcher. It included items of ' 'mother's preferences in labor, mobility, eating and drinking, monitoring, pain relief, drug options, labor augmentation, pushing, amniotomy, episiotomy, infant care, and C-section. Routine hospital care (*e.g.,* continuous monitoring of fetal heart rate, control of labor progress, etc*.*) was provided to members of the control group.

### Scales and data collection

The data were collected using the socio-demographic and obstetrics characteristics questionnaire, Birth Plan Checklist, W-DEQ-version A, EPDS, Childbirth Experience Questionnaire 2.0 (CEQ 2.0), Support and Control in Birth (SCIB) scale, partograms, Delivery Fear Scale (DFS), PTSD Symptom Scale (PSS), and Maternal and Neonatal Outcomes Checklist. The W-DEQ-version A and EPDS were completed at the beginning of the study (at the gestational age of 32–36 weeks). In addition, the maternal and neonatal information was collected both during and immediately after the delivery, and the researcher completed the partograms and DFS during labor for all the participants. The participants in both groups were followed up 4–6 weeks after delivery. The researcher completed the CEQ 2.0, SCIB, EPDS, and PSS 4–6 weeks after delivery by contacting the studied mothers via telephone (due to the outbreak of COVID-19). A figure showing the development of the intervention and which questionnaires were used in each step is avialable as Appendix 1.

### Socio-demographic characteristics questionnaire

This questionnaire contained items about age, level of education, occupation of participant and her spouse, marital status, marriage age, residence status, household income, pre-pregnancy weight, and height.

### Obstetric characteristics questionnaire

This questionnaire included items about the number of pregnancies, number of parity, number of abortions, history of infertility, participation in childbirth preparation classes, and pregnancy type in terms of planned or unplanned.

### Birth plan checklist

Kitzinger put forward the idea of the birth plan in the US in the 1980s [21]. This includes women's preferences in labor, mobility, eating and drinking, monitoring, pain relief, pharmacological options, acceleration of labor, pushing, amniotomy, episiotomy, care of the child, and cesarean section. It is completed during pregnancy in consultation with healthcare staff or obstetricians by pregnant women. The birth plan was rapidly executed in some European countries and is used in 78% of delivery rooms in England [22]. The birth plan checklist is available as appendix 2.

To determine the validity of the birth plan checklist, we used the questionnaires translation, content, and face validity. Specifically, the checklist was evaluated by ten specialists. After collecting their opinions, the necessary corrections were made to the checklist based on the feedback acquired.

### Childbirth experience questionnaire (CEQ 2.0)

This instrument measured the childbirth experience for women and contains 25 items. The questionnaire covers the following areas: professional support (midwifery care and information), perceived security (sense of security and memories of childbirth), personal capacity (locus of control is a psychological concept that refers to how strongly people believe they have control over the situations and experiences that affect their lives, personal feelings about childbirth and labor pain), and participation (the person's ability to change the position, movements, and pain mitigation during labor). Specifically, three items are completed based on a visual analog scale (VAS), the items responded based on VAS are changed into values 1– 4: scores 0–40 (score 1), scores 41–60 (score 2), scores 61–80 (score 3), and scores (81–100) (score 4), and 23 items are multiple-choice (with four options), the responses are in the form of absolutely agree (score 4), often agree (score 3), often disagree (score 2), and absolutely disagree (score 1). Sentences with negative concepts (experience of severe pain, sense of fatigue, fear, and having bad memory) are scored negatively. This instrument's high mean values represent a more positive childbirth experience [23]. Also, the reliability and validity of the Persian version of this questionnaire have been determined in the research setting by Ghanbari Homaei et al. The Cronbach alpha coefficient of items and the intra-class correlation coefficient was reported to be 0.93 and 0.97, respectively [24].

### Support and Control in Birth (SCIB)

This scale contains 33 items, and its subscales include internal control, external control, and support. Internal control contains ten items that assess pain, emotions, and behavior (such as: I overcame my pain); External control contains 11 items that focus on decisions and procedures (e.g., I had control when the procedure was performed), and support includes 12 items that focus on attitude, patience, empathy, and coping with pain (e.g., caregivers Ignored what I wanted) (Ford et al., 2009). The questions are graded on a Likert scale of 5 points from Strongly Agree to Strongly Disagree [25]. Score 1 indicates less control and support, and score 5 indicates more control and support. Items 1, 2, 6, 7, 10, 14, 17, 28, 29 and 33 are scored negatively. Instrument psychometrics in Iran has been performed by Ghanbari et al., and Cronbach's alpha of the items has been reported between 0.86 and 0.93 [26].

### Delivery Fear Scale (W-DEQ-Version A)

In order to assess the fear of childbirth during pregnancy, W-DEQ-Version A was used. This questionnaire, first designed by Wijma et al. in 1998, measures fears and expectations related to delivery with 33 items. The mothers express their personal emotions and perceptions based on a 6-point Likert scale (zero = never; 1 = very Low; 2 = Low; 3 = Average; 4 = high; 5 = very high). Generally, the total score is obtained by summing up the score of all items, with scores ranging between 0 and 165. Scores equal to or less than 37, 38–65, 66–84, and greater than 85 represent low, moderate, high, and intense fear. Wijma et al. estimated the reliability of the questionnaire through the split-half method and the Cronbach alpha coefficient as 0.89 and 0.92, respectively. (Wijma et al., 1998) [27]. The reliability and validity of this questionnaire have been determined by Mortazavi (2017) in Iran, with a Cronbach alpha coefficient of 0.91 [28].

### Delivery Fear Scale (DFS)

In order to assess the fear during labor, the DFS designed by Wijma et al. (2002) was used. DFS is a valid 10-item self-assessment questionnaire, capturing the fear of delivery during labor through scores ranging from 1 = absolutely disagree to 10 = absolutely agree. The range of scores is 10–100. Higher scores represent greater fear. The Persian version of DFS is a reliable and valid tool to measure fear in the delivery room in the active phase of labor [29]. Cronbach's alpha of this questionnaire in the study by Shakarami et al. has been calculated 0.77 [30].

### Edinburgh’s postpartum depression scale (EPDS)

This questionnaire was used to measure postnatal depression as well as depression during pregnancy, first developed by Cox et al. in 1987. This instrument consists of 10 multiple-choice questions (with four options). The options for each item claim a score from 0 to 3 based on the severity of symptoms. In some items, the choices are ordered from low to high (items 1, 2, and 4), while in other cases, from high to low (items 3, 5, 6, 7, 8, 9, 10). The range of scores is 0–30. The validity of this scale using the method of determining the concurrent correlation coefficient was calculated to be 0.78. Also, the reliability using the Cronbach alpha method and split-half method was estimated as 0.75 [31]. Montazeri et al. reported the Cronbach alpha value associated with the postnatal period as 0.77, with the intra-class correlation coefficient of 0.80 [32].This questionnaire was completed through an interview by the researcher when the mother was referred to routine checkups during 32–36 weeks of gestation. The mothers acquiring scores above the threshold limit of 12 have depression with different severity. Also, six weeks into postpartum, we completed the questionnaire again via an interview to examine postnatal depression. It should be noted that pregnant women with EPDS scores ≥ of 13 were referred to a psychiatrist.

### PTSD symptom scale 1 (PSS-I)

It includes 17 items that completely cover all criteria of the fourth version of the diagnostic and statistical manual of psychiatric disorders (DSM-IV) to diagnose post-traumatic stress disorder and marks. The indicators of this disorder include symptoms associated with re-experiencing (four items), symptoms related to avoidance (seven items), and symptoms that pertain to arousal (6 items). In the case of having one or more symptoms of re-experiencing and three or more symptoms related to avoidance and two or more factors related to arousal, a PTSD diagnosis is made. The severity of symptoms of every criterion using a Likert scale. The range of scores is 0–51. The Cronbach alpha of the Persian version of the was 0.88, and the Kappa coefficient calculated with test–retest methods has been reported as 1 [33].

### Partogram form

Partogram is a valid diagram, simple and inexpensive, which is indeed the best instrument for monitoring the process of delivery as well as maternal and neonatal health. It involves registering information regarding the maternal health status, fetal health status, recording the process of delivery, and managing the delivery. It allows healthcare staff to express the details of delivery visually. Indeed, it is an early warning system that remarkably helps in decision-making on the timely referral of the mother [34].

### Data analysis

The data were analyzed in SPSS-version 22. Descriptive statistics of frequency, percentage, mean, and standard deviation (SD) were used s to describe the participants' socio-demographic and obstetrics characteristics. The Kolmogorov–Smirnov (K-S) test was used to assess the normality of the quantitative data, and all the data had normal distributions. All the analyses were based on intention to treat. The independent t-test was used to compare the study groups in terms of the variables of the childbirth experience, support, and control in birth, pre-intervention depression and fear, post-traumatic stress disorder, duration of the active, second, and third phases of labor (minutes), and the first and fifth minute Apgar scores. The chi-square test was used to compare the study groups in terms of the type of delivery and frequency of admission of a newborn in NICU. Finally, the ANCOVA with adjusting the baseline values was used to compare the variables of fear of delivery (during labor) and postnatal depression among groups.

## Results

**Fig. 1:**
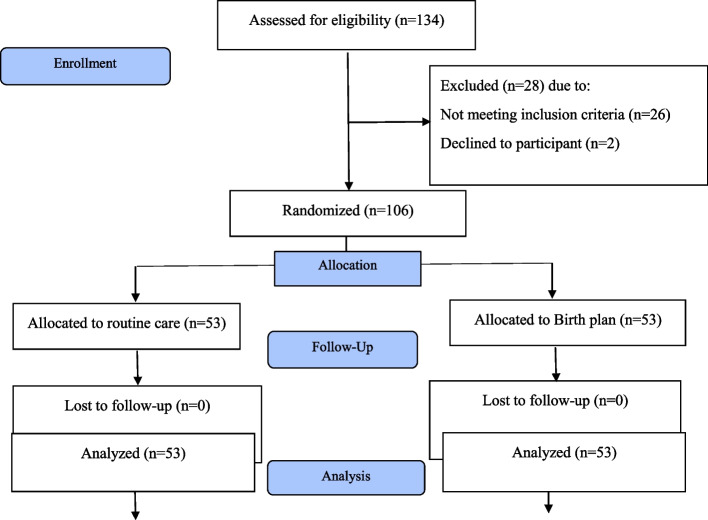
Flow diagram of study

The sampling process began in late October 2020 and ended in late June 2021. The researcher assessed 134 pregnant mothers, among whom 26 individuals were excluded due to high-risk pregnancy (diabetes, heart disease, and abnormal fetus), and two others were excluded due to unwillingness to participate in the study; therefore, 106 eligible individuals were selected as the sample. No case of loss to follow-up was observed, and all the mothers were followed up 4–6 weeks after delivery (Fig. 1).

The mean (SD) age of the participants in the intervention and control groups was 25.1 (5.3) and 26.8 (5.4) years, respectively. Table 1 shows the other socio-demographic of participants.

**Table 1:** Socio-demographic characteristics of the participants

Characteristics	Birth plan group (*n* = 56)	Control group (*n* = 56)	*P*-Value
	Mean (SD)	Mean (SD)	
**Age** (Year)	25.1 (5.3)	26.8 (5.4)	0.105^†^
**Husband's Age** (Year)	30.2 (51)	31.4 (4.6)	0.226^†^
**Married age** (Year)	19.2 (4.9)	21.1 (5.8)	0.078^†^
**Gestational age** (Week)	39.3 (0.8)	39.5 (0.7)	0.156^†^
	Number (Percent)	Number (Percent)	
**Education**			0.076^§^
Under diploma	38.0 (71.7)	27 (50.9)	
Diploma	11.0 (20.8)	16.0 (30.2)	
University	4.0 (7.5)	10.0 (18.9)	
**Spouse education**			0.067^§^
Under diploma	39.0 (73.6)	28.0 (52.8)	
Diploma	11.0 (20.8)	17.0 (32.1)	
University	3.0 (5.7)	8.0 (15.1)	
**Job**			0.716^§^
Housewife	50.0 (94.3)	48.0 (90.6)	
Working at home	3.0 (5.7)	5.0 (9.4)	
**Spouse employment**			0.381^‡^
Unemployed	1.0 (1.9)	0.0 (0.0)	
Employee	4.0 (7.5)	4.0 (7.5)	
Manual worker	11.0 (20.8)	8.0 (15.1)	
Self-employment	37.0 (69.8)	41.0 (77.4)	
**Home status**			1.000^§^
Private house	38.0 (71.7)	37.0 (69.8)	
Rented house	15.0 (28.3)	16.0 (30.2)	
**Sufficiency of income for expenses**			0.737^‡^
Insufficient	1.0 (1.9)	1.0 (1.9)	
Somewhat sufficient	48.0 (90.6)	49.0 (92.5)	
Completely sufficient	4.0 (7.5)	3.0 (5.7)	
**Body mass index** (kg/m^2^)			0.124^§^
18.5 ≥	4.0 (7.5)	6.0 (11.3)	
18.5 to 24.9	29.0 (54.7)	18.0 (34.0)	
25.0 to 29.9	14.0 (26.4)	24.0 (45.3)	
≥ 30	6. (11.3)	5.0 (9.5)	0.346^‡^
**Gravid**			
1	25.0 (47.2)	22.0 (41.5)	
2	23.0 (43.4)	24.0 (45.3)	
3	5.0 (9.4)	5.0 (9.4)	
4	0.0 (0.0)	2.0 (3.8)	
**Number of parity**			0.331^§^
1	31.0 (58.5)	25.0 (47.2)	
2	22.0 (41.5)	28.0 (52.8)	
**History of abortion**			1.000^¥^
No	44.0 (83.0)	44.0 (83.0)	
Yes	9.0 (17.0)	9.0 (17.0)	
**History of infertility**			1.000^§^
No	51.0 (96.2)	51.0 (96.2)	
Yes	9.0 (17.0)	9.0 (17.0)	
**Type of Pregnancy**			1.000^§^
Planned	50.0 (94.3)	50.0 (94.3)	
Unplanned	3.0 (5.7)	3.0 (5.7)	
**Attending in childbirth preparation classes**			1.000^§^
No	53.0 (100.0)	52.0 (98.1)	
Yes	0.0 (0.0)	1.0 (1.9)	

^‡^Chi-square for trend test; ^§^Fisher's exact test; ^¥^Chi-Square test; ^†^Independent t-test

The mean (SD) CEQ score (Mean Difference (MD): 1.0; 95% Confidence Interval (95% CI): 1.1 to 0.9; P˂0.001) and SCIB score (MD: 53.9; 95% CI: 57.4 to 50.4; *P*˂0.001) of the participants were significantly higher in the birth plan group compared to the control group.

The results of ANCOVA with adjusting the baseline values showed that the mean score of fear of childbirth during labor (Adjusted Mean Difference (AMD): -5.5; 95% CI: -9.9 to -1.1; *P* = 0.015) and postpartum depression (AMD: 4.8; 95% CI: 3.9 to 5.7; P˂0.001) in the birth plan group were significantly lower than those in the control group. In addition, the mean (SD) PTSD score of the participants in the birth plan group was significantly lower than that of those in the control group (MD: 8.8; 95% CI: 10.2 to 7.4; P˂0.001) also, in subgroup analysis by parity indicated no significant difference between the birth plan and control groups (Table 2).

**Table 2:** Comparison of childbirth experience, support and control in birth, fear of childbirth, postpartum depression, and postpartum stress disorder among the study groups

Variable	Birth plan (*n* = 56)	Control (*n* = 56)	MD (95% CI)^a^	*P*-Value
	Mean (SD^b^)	Mean (SD^b^)		
**Childbirth experience** (Score range: 1 to 4)	3.2 (0.2)	2.1 (0.2)	1.0 (1.1 to 0.9)	˂0.001^*^
**Primiparous**	3.1 (0.2)	2.1 (0.2)	1.0 (1.2 to 0.9)	˂0.001^*^
**Multiparous**	3.2 (0.2)	2.1 (0.2)	1.0 (1.2 to 0.9)	˂0.001^*^
**Support and Control in Birth** (Score range: 31 to 155)	123.5 (11.0)	69.5 (6.7)	-53.9 (-57.4 to -50.4)	˂0.001 ^*^
**Primiparous**	123.5 (10.7)	69.5 (6.0)	-53.7 (-58.5 to -48.9)	˂0.001 ^*^
**Multiparous**	123.7 (11.7)	69.6 (7.3)	-54.1 (-59.5 to -48.7)	˂0.001 ^*^
**Fear of childbirth during pregnancy** (Score range: 0 to 165)	121 (20.0)	117 (21.0)	-3.7 (-11.7 to 4.1)	0.348 ^*^
**Primiparous**	118.6 (19.5)	113.2 (22.5)	-5.4 (-16.9 to 6.0)	0.344 ^*^
**Multiparous**	124 (20.6)	121 (19.3)	-3.5 (-15.0 to 8.0)	0.548 ^*^
**Fear of childbirth in labor** (Score range: 10 to 100)	28.3 (13.0)	25.1 (14.1)	5.5 (1.1 to 10.0)	0.015^†^
**Primiparous**	31.5 (14.9)	38.7 (17.8)	4.6 (2.4 to 11.6)	0.192^†^
**Multiparous**	23.8 (8.2)	31.9 (8.7)	7.6 (3.0 to 12.1)	0.002^†^
**Depression before intervention** (Score range: 0 to 30)	2.6 (1.8)	2.9 (1.9)	0.3 (1.0 to -0.4)	0.441 ^*^
**Primiparous**	2.7 (2.0)	3.1 (2.2)	0.4 (1.6 to -0.7)	0.430 ^*^
**Multiparous**	2.6 (1.7)	2.8 (1.7)	0.3 (1.1 to -0.8)	0.757 ^*^
**Postpartum depression** (Score range: 0 to 30)	2.9 (1.8)	7.7 (2.8)	4.8 (3.9 to 5.7)	˂0.001^†^
**Primiparous**	2.7 (1.6)	7.6 (2.8)	4.8 (6.0 to 3.6)	˂0.001^†^
**Multiparous**	3.1 (2.1)	7.9 (2.8)	4.7 (3.2 to 6.2)	˂0.001^†^
**Post-traumatic stress disorder** (Score range: 0 to 51)	2.5 (2.9)	11.3 (4.3)	8.8 (10.2 to 7.4)	˂0.001^*^
**Primiparous**	2.5 (3.0)	12.2 (4.6)	9.8 (7.6 to 11.9)	˂0.001^*^
**Multiparous**	2.6 (2.8)	10.5 (4.3)	7.8 (5.9 to 9.8)	˂0.001^*^

^*^Independent t-test; ^†^ANCOVA; ^a^Mean Difference (95% Confidence Interval); ^b^Standard Deviation

The independent t-test results indicated no significant difference between the birth plan and control groups in terms of the mean duration of the active phase of labor (MD: -14.3; 95% CI: -37.5 to 8.9; *P* = 0.223), the second phase of labor (MD = -0.5; 95% CI: -8.5 to 7.5; *P* = 0.903) and the third phase of labor (MD = -0.8; 95% CI: -1.0 to 2.6; *P* = 0.386).

In total, three mothers (5.7%) in the birth plan group (two cases due to fetal heart rate deceleration and one case due to arrest of cervical dilation) and 14 mothers (26.4%) in the control group (eight cases due to fetal heart rate deceleration, four cases due to arrest of cervical dilation and one case due to arrest of descent) underwent emergency C-sections, and the difference was significant based on Fisher's exact test results (*P* = 0.007). In both groups, three infants were admitted to NICU, and Fisher's exact test results showed no difference between the two groups (*P* = 1.000). Also, the mean (SD) of neonatal Apgar score in the first minute in the birth plan group was significantly higher than those in the control group. (*P* = 0.048). No statistically significant difference was observed between the birth plan and control groups regarding the mean (SD) fifth-minute Apgar scores of infants (*P* = 0.731) (Table 3).

**Table 3:** Comparison of delivery outcomes (maternal and neonatal) among the study groups

Variable	Birth plan (*n* = 56)	Control (*n* = 56)	MD (95% CI)^a^	*P*-Value
	Mean (SD^b^)	Mean (SD^b^)		
**Duration of labor stages**				
**Active phase (Minute)**	222.0 (57.0)	207.7 (53.4)	-14.3 (-37.5 to 8.9)	0.223^*^
**Primiparous**	237.7 (59.4)	215.6 (55.6)	-22.1 (-58.6 to 14.3)	0.228^*^
**Multiparous**	200.2 (46.6)	202.6 (52.5)	2.4 (-27.1 to 31.8)	0.^*^0872
**Second stage (Minute)**	41.9 (12.5)	41.4 (24.6)	-0.5 (-8.5 to 7.5)	0.903^*^
**Primiparous**	45.3 (12.6)	55.4 (0.4)	-10.0 (-5.3 to 25.3)	0.194^*^
**Multiparous**	37.1 (11.0)	33.6 (6.0)	-3.5 (-9.0 to 1.9)	0.198^*^
**Third stage (Minute)**	5.7 (1.7)	6.5 (6.1)	0.8 (-1.0 to 2.6)	0.386^*^
**Primiparous**	5.4 (1.4)	8.7 (9.8)	3.3 (-2.4 to 9.0)	0.084^*^
**Multiparous**	6.0 (2.0)	5.2 (1.0)	0.8 (-1.7 to 0.1)	0.107^*^
**Apgar score of the first minute**	9.0 (0.4)	8.8 (0.6)	-0.2 (-0.4 to 0.0)	0.048^*^
**Primiparous**	8.9 (0.4)	8.6 (0.8)	-0.4 (-0.7 to 0.3)	0.033^*^
**Multiparous**	9.0 (0.3)	8.9 (0.4)	0.7 (0.3 to 0.1)	0.0476^*^
**Apgar score of the five minute**	9.9 (0.3)	9.9 (0.3)	-0.2 (-0.1 to 0.1)	0.731^*^
**Primiparous**	9.9 (0.3)	9.9 (0.3)	-0.2 (-0.2 to 0.1)	0.785^*^
**Multiparous**	9.9 (0.2)	9.9 (0.3)	-0.2 (-0.2 to 0.1)	0.708^*^
	Number (Percent)	Number (Percent)	Odds ratio (95% CI)	
**Mode of Delivery in all participants**			0.17 (0.05 to 0.62)	0.007^§^
Normal vaginal delivery (spontaneous) (NVD)	50 (94.3)	39 (73.6)		
Emergency cesarean section	3 (5.7)	14 (26.4)		
**Primiparous**			0.08 (0.02 to 0.45)	0.001^¥^
Normal vaginal delivery	14 (56.0)	29 (93.5)		
Emergency cesarean section	11 (44.0)	2 (6.5)		
**Multiparous**			0.40 (0.04 to 4.10)	0.621^§^
Normal vaginal delivery	25 (89.3)	21 (95.5)		
Emergency cesarean section	3 (10.7)	1 (4.5)		
**Admission of a newborn in NICU in all participants**			1.0 (0.19 to 5.20)	1.000^§^
No	50 (94.3)	50 (94.3)		
Yes	3 (5.7)	3 (5.7)		
**Primiparous**			0.51 (0.08 to 3.29)	0.647^§^
No	22 (88.0)	29 (93.5)		
Yes	3 (12.0)	2 (6.5)		
**Multiparous**			-	0.440^§^
No	28 (100)	21 (95.5)		
Yes	0	1 (4.5)		

^*^Independent t-test; ^§^Fisher's exact test; ^a^Mean Difference (95% Confidence Interval); ^b^Standard Deviation; ^¥^Chi-square

## Discussion

Birth plans improved childbirth experiences of mothers, as well as their perceived control and support during labor. It also reduced fear of childbirth, postpartum depression symptoms, and post-traumatic stress symptoms and increased the frequency of vaginal delivery and neonatal Apgar scores in the first minute. However, there was no significant difference between the two groups in terms of duration of active, second, and third phases of labor, fifth minute Apgar scores, and frequency of admission of a newborn in NICU. This was the first study to investigate the effects of the birth plan on childbirth experiences and maternal and neonatal outcomes in Iran.

In this study, the mean CEQ score of the participants in the birth plan group was significantly higher than that of those in the control group. In a quasi-experimental study in Egypt, Farahat et al. (2015) found that women's mean childbirth experience score in the intervention group was significantly higher than those in the control group. In addition, the satisfaction scores of women in the intervention group were significantly higher than those in the control group both during and after delivery [35]. However, a systematic review by Mirghafourvand et al. (2019) found that there was insufficient evidence to support or refute that birth plans can improve the birth experience or satisfaction with birth [17]. Childbirth is one of the most challenging psychological events in a mother's life. Providing continuous support and paying attention to the needs of mothers during labor are the main determinants of childbirth experience [36]. A good midwife-mother relationship can help mothers undergo positive childbirth experiences by reinforcing the perceived control of mothers over childbirth events and processes. It also enables mothers to examine different issues and choose the best birth options. Midwives and other health care providers dramatically influence the childbirth experiences of mothers [37]. Of course, the availability of pain relief measures and the possibility of combining pharmacological and non-pharmacological methods be effective factors in childbirth experiences of mothers [38].

In the present study, the support and control (SCIB) score of the women in the birth plan group was significantly higher than that of those in the control group. Kuo et al*.* conducted a clinical trial to examine the effects of the birth plan on childbirth experiences, sense of control, and expectations of 330 primigravid women with a minimum gestational age of 32 weeks. The participants were selected from 7 hospitals and ten obstetric clinics in Taiwan. Based on their findings, positive childbirth experiences and perceived control and support of women in the birth plan group were significantly higher than those in the control group [39]. Support and communication during labor increase a "woman's satisfaction with childbirth". This effective communication should be established upon admission and improved continuously during labor. The provision of relevant information to pregnant mothers can boost their active participation in the decision-making process [28]. Satisfaction with childbirth reflects a good feeling about giving birth, which originates from a sense of participation and control, fulfillment of needs and expectations, power, empowerment, and support [39, 40]. Research shows that birth experiences are determined by a range of factors, including support, control, internal control, and obstetric outcomes [41, 42]. Theoretical and empirical evidence suggests that control and support are potentially related to childbirth outcomes. Accordingly, lack of control and support during labor is associated with anxiety, PTSD, and depression [41, 43–45].

In this study, the mean childbirth fears score of the mothers in the birth plan group was significantly lower than that of those in the control group. This result is consistent with the findings of Lundgren et al*.* [46]. Evidence demonstrates that fear of childbirth, self-confidence (self-efficacy), and sense of control are closely associated together [47]. Moreover, environmental factors and women's interaction with obstetric care staff affect their fear of childbirth. Women who experience a terrible fear of childbirth are worried about their performance and body's ability during labor. These worries are associated with poor expectations of positive results and poor coping abilities [48]. Therefore, pregnant mothers can significantly reduce their fear of childbirth by interacting with health professionals, receiving necessary training about labor, obtaining information about different birth options, and developing strategies for a positive birth experience [49, 50].

In the present study, the mean depression symptoms and post-traumatic stress symptoms scores of the participants in the birth plan group were significantly lower than those obtained for mothers in the control group. In a mixed-methods study, Benoit et al*.* (2007) concluded that dissatisfaction with childbirth experience is closely related to postpartum depression. Women who developed birth plans and received ongoing support from midwives enjoyed a better childbirth experience and were less likely to experience postpartum depression. In addition, this positive feeling had a lasting positive effect on the mother-infant relationship [40]. According to studies, patient-centered care improves outcomes in areas such as self-care, patient satisfaction, and adherence to treatment [51, 52]. Labor leads to several short-term and long-term psychological outcomes for mothers; accordingly, negative birth experiences are associated with post-traumatic stress disorder and depression [53–56]. No researcher has so far thoroughly investigated the effect of the birth plan on post-traumatic stress.

In this study, the prevalence of emergency C-sections was lower in the birth plan group than in the control group. Also, the mean of neonatal Apgar score in the first minute in the birth plan group was significantly higher than those in the control group. However, no significant differences were observed between the two groups in terms of the duration of the active, second, and third phases of labor, frequency of admission of a newborn in NICU, and fifth minute Apgar scores. Between 2011 and 2012, Suárez-Cortés et al*.* carried out a descriptive cross-sectional study in the border areas of Spain on 9303 women to compare childbirth outcomes of the birth plan group with those of the control group. Only 2.6% of the women (240 individuals) had birth plans. Significant differences were found between those with and those without a birth plan in terms of skin-to-skin contact, selection of labor position, and shaving [22]. A retrospective study was conducted in a military hospital in the United States (*n* = 67). The results showed that 75% of women had spontaneous vaginal delivery, 6% had an operative vaginal delivery, and 19% had C-section [57]. The same author conducted another study in 2007 to compare the frequency of C-sections in the birth plan and control groups. Based on their findings, 17% of people in the birth plan group and 12% of those in the control group had C-sections [58]. In Hidalgo-Lopezosa et al. study, no significant difference was found between the birth plan (24.3%) and the control (27%) groups in terms of frequency of C-sections [59]. The patient is the best source of information on his or her own condition. The challenge for healthcare professionals is to encourage people not engaged in their own care to become involved and help the engaged those to stay engaged [60]. Researches indicate that people who remain engaged in their own health care are more likely to remain healthy. The engaged person is interested in his or her own medical conditions and stay abreast of information to promote their health [12, 61].

Hidalgo-Lopezosa et al. conducted a case–control study in Spain from 2008 to 2011 to compare maternal and neonatal outcomes of birth plans. To this end, they assigned 52 women to the birth plan group and 130 individuals to the control group. Furthermore, there was no statistically significant difference between the two groups in terms of the prevalence of episiotomy, 3rd–4th-degree tear, and spontaneous labor; however, the prevalence of umbilical cord arterial blood pH < 24.7 was significantly lower in the intervention group than in the control group [59]. Hidalgo-Lopezosa et al. carried out a prospective descriptive study on 178 pregnant women in a third-level hospital in Spain between 2009 and 2013, where the mothers birth plan was routinely implemented. Based on the results, 73% of the women had spontaneous onset of labor; 27% had induced labor, and 43% underwent episiotomy, 34% underwent amniotomy, and 70% received epidural anesthesia. In 24% of mothers, fetal heart rate was monitored intermittently (freedom of movement). In 76% of the participants, fetal heart rate was monitored continuously (68% of them were monitored externally; 8% were monitored internally) [62]. The WHO recommends the implementation of the birth plan, and promotes the use of more natural processes. This organization also disapproves of the excessive use of medical procedures and interventions such as episiotomy, early amniotomy, and routine use of oxytocin, lithotomy position, and continuous monitoring of fetal heart rate [63].

'Mothers' training and their participation in the decision-making process substantially affect their physical and psychosocial preparation for labor. Decision-making participation is a process in which the therapist and patient work together to enhance the treatment process using the best available scientific evidence [64]. Scholars have paid more attention to 'patients' values, preferences, and desires over the past few decades. In addition, services have been primarily focused on improving the level of care and patient-centered care [65]. Birth plans are devised to involve women in labor and delivery decisions. Care systems can enhance care levels by increasing 'patients' involvement in their care procedures [66].

### Strengths and limitations

This was the first study to assess the implementation of a birth plan in Iran. The participants consisted of both multiparous and nulliparous women living in a metropolis in Iran; therefore, the results can be generalized to nulliparous and multiparous women residing in other similar environments and cities. In addition, the large size of the study sample and the no loss to follow-up rate added to the credibility of the findings. Using standandard tools validated in Iran was another stength of the study. Also, due to the close and frequent follow-up of participants, there was no loss.

Due to the outbreak of COVID-19, women in the birth plan group attended the training sessions separately. Finally, in the postpartum stage, the questionnaires were completed by contacting the participants via telephone. These deviations from the designed protocol were among the weaknesses of the study. Completion of partogram by the researcher was another limitation of the present study. Also, postpartum depression in this study was measured 4 to 6 weeks after delivery, while postpartum depression may occur with a longer interval after delivery, so some cases may not be diagnosed. Since the principal investigator attended the women on the day of delivery, this may be as a possible bias.

## Conclusion

Based on the findings, a birth plan improves childbirth experiences; increases perceived support and control in labor; reduces fear of childbirth; suppresses psychological symptoms of depression and PTSD, increases the frequency of vaginal delivery, and neonatal Apgar scores in the first minute. However, the plan had no significant impact on the duration of labor phases, neonatal Apgar scores neonatal in the fifth minute, and frequency of admission of a newborn in NICU. The present findings can be used to improve relevant educational, managerial, policy, and clinical decisions. A birth plan can raise awareness among mothers and increase their control, support, and participation in decisions made during the labor and delivery processes, and thereby improve their childbirth experience. 'Mothers' satisfaction with the childbirth experience is among the most important indicators of maternal care quality. The following practices are suggested to increase 'mothers' satisfaction with the labor process and improve maternal experiences and outcomes: raising awareness among providers of prenatal, labor, and delivery services, and encouraging them to use the birth plan; policy-making and designing standard birth plans in hospitals to enhance obstetric care; evaluating the implementation of the birth plan; providing relevant staff with necessary training materials, and obliging the presence of a skilled midwife in the labor and delivery processes.

## Supplementary Information

**Additional file 1:** **Supplementary figure**.  The used questionnaires in each step of study

**Additional file 2**. 

## Data Availability

The datasets generated and/or analysed during the current study are not publicly available due to limitations of ethical approval involving the patient data and anonymity but are available from the corresponding author on reasonable request.

## Abbreviations

PTSD: Post-Traumatic Stress Disorder
WHO: World Health Organization
W-DEQ-version A: Wijma Delivery Expectancy/Experience Questionnaire-version A
EPDS: Edinburgh Postnatal Depression Scale
CEQ 2.0: Childbirth Experience Questionnaire 2.0
SCIB: Support and Control in Birth
DFS: Delivery Fear Scale
COVID-19: Coronavirus Disease of 2019
VAS: Visual Analog Scale
PSS-I: PTSD Symptom Scale 1
DSM-IV: Diagnostic and Statistical Manual of Psychiatric Disorders
SD: Standard deviation
K-S: Kolmogorov–Smirnov
NICU: Neonatal Intensive Care Unit
ANCOVA: Analysis of Covariance
AMD: Adjusted Mean Difference
