# Supplementary material for: Effect of implementing a birth plan on maternal and neonatal outcomes: a randomized controlled trial
Source: BMC Pregnancy Childbirth. 2022 Nov 22;22:862. doi: 10.1186/s12884-022-05199-5 (PMC9682672; doi:10.1186/s12884-022-05199-5)
Supplement: Supplementary file 1 — Additional file 1: Supplementary figure. The used questionnaires in each step of study [file 12884_2022_5199_MOESM1_ESM.docx]

Written informed consent was obtained (n= 106)

Assessed for eligibility and completed Edinburgh Postnatal Depression Scale (EPDS) (n= 134)

**Supplementary figure:** The used questionnaires in each step of study

Follow-up of both groups 4-6 weeks after delivery, and completion of CEQ 2.0, SCIB, EPDS, and PSS 4-6 weeks after delivery by contacting the studied mothers via telephone (due to the outbreak of COVID-19).

Completion of Partogram, Delivery Fear Scale (DFS), and Maternal and Neonatal Outcomes Checklist for all participants by researcher

Implementing a birth plan for birth plan group with the entrance of the mothers to the labor ward

Allocated to routine care (n=53)

Holding a training session for the members of the birth plan group and preparing the birth plan

Allocated to Birth plan (n=53)

Random allocation (n= 106)

Completion of the socio-demographic and obstetrics characteristics questionnaire, the Wijma Delivery Expectancy/Experience Questionnaire-version A (W-DEQ-version A) (n= 106)
