# Supplementary material for: Effect of implementing a birth plan on maternal and neonatal outcomes: a randomized controlled trial
Source: BMC Pregnancy Childbirth. 2022 Nov 22;22:862. doi: 10.1186/s12884-022-05199-5 (PMC9682672; doi:10.1186/s12884-022-05199-5)
Supplement: Supplementary file 2 — Additional file 2. [file 12884_2022_5199_MOESM2_ESM.docx]

**Birth Plan: Checklist**

Fill out this page according to your own wishes for your birth. Keep in mind that you might not be able to follow every wish on this page depending on hospital policy or if complications arise during your labor. Share your plan with your support team, practitioner, and midwife.

**Labor**

○ Dim Lighting ○ Play Music ○ Quiet

○ Bring things in from home like blankets or photos ○ Aromatherapy scents

○ Playing my favorite music ○ I want .............. to be with me in labor.

**Hydration and Nourishment**

○ I would like to eat light like soup in labor.

○ I would like to drink liquids in labor.

○ I wouldn't like to eat and drink in labor.

**Mobility**

○ I prefer to maintain all mobility, including walking and changing positions

○ I want to use bedside foam for all fours, sitting, and exercising.

○ I prefer to lie on the bed and rest.

**Monitoring**

○ I prefer the fetus to be regularly monitored (fetal heart examination).

○ I prefer to monitor the fetal heart rate intermittently (every 15 minutes in the first stage and every 5 minutes in the second stage between contractions) at the discretion of the doctor.

**Pain relief**

**Nonmedical Options**

○ Relaxation ○ Changing positions/walking ○ Intellectual visualization

○ Massage ○ Fitness ball ○ Breathing

○ Hot shower ○ Hot and cold packs

**Medical Options**

○ I like to use intramuscular and intravenous pain relievers for labor pain relief.

○ I don't like any painkillers to be used to relieve labor pain.

**Augmentation**

**Methods to Speed Up Labor**

○ I prefer not to use oxytocin in any way to speed up my labor.

○ I prefer, at the doctor's discretion, to use oxytocin to speed up my labor if needed.

○ I prefer to continue to use oxytocin to speed up my labor despite the presence of active uterine contractions.

**Amniotomy**

○. Prefer that my practitioner or midwife breaks my bag of water.

○ Prefer that my bag of water breaks on its own.

I don't have any opinion about the rupture of the bag of water.

**Pushing**

○ I would like to push whenever I feel like it.

○ I would like to be directed as to when to push.

○ I prefer to wait to push until I feel the urge or until my baby descends.

**Episiotomy**

○ I prefer not to have an episiotomy.

○ I would like to have an episiotomy at the discretion of the doctor or midwife.

**Child birth**

○ I like to touch the baby's head while crooning. Yes ◯ No ◯

○ I would like a mirror placed at the foot of the bed so I can watch my baby’s birth. Yes ◯No◯

**Child care**

○ I don't like to breastfeed immediately after childbirth.

○ I like to breastfeed immediately after childbirth.

○ I would like the routine hospital procedure to be followed for my baby.

**In cases of caesarean section**

○ I would like midwife to accompany me during surgery.

○ I like my accompanying midwife to explain the surgical procedures to me.

○ I would like to breastfeed in the recovery room soon.
